# Electrochemical Biosensors: A Solution to Pollution Detection with Reference to Environmental Contaminants

**DOI:** 10.3390/bios8020029

**Published:** 2018-03-24

**Authors:** Gustavo Hernandez-Vargas, Juan Eduardo Sosa-Hernández, Sara Saldarriaga-Hernandez, Angel M. Villalba-Rodríguez, Roberto Parra-Saldivar, Hafiz M. N. Iqbal

**Affiliations:** 1 Tecnologico de Monterrey, School of Engineering and Sciences, Campus Monterrey, Ave. Eugenio Garza Sada 2501, CP 64849, Monterrey, N.L., Mexico; gushdzv@gmail.com (G.H.-V.); eduardo.sosa@itesm.mx (J.E.S.-H.); sarasaldarriaga.h@gmail.com (S.S.-H.); angel.vr@itesm.mx (A.M.V.-R.); 2 Exact and Natural Sciences, Institute of Biology, University of Antioquia, St. 67 No. 53-108, Medellín 050021, Colombia; 3 Microsystems Technologies Laboratories, Massachusetts Institute of Technology (MIT), Cambridge, MA 02139, USA; 4 Division of Engineering in Medicine, Department of Medicine, Brigham and Women’s Hospital, Harvard Medical School, Cambridge, MA 02139, USA; 5 Harvard-MIT Division of Health Sciences and Technology, MIT, Cambridge, MA 02139, USA

**Keywords:** electrochemical biosensors, screen-printed electrodes, nanowire sensors, paper-based biosensors, emerging contaminants, toxic heavy elements, detection

## Abstract

The increasing environmental pollution with particular reference to emerging contaminants, toxic heavy elements, and other hazardous agents is a serious concern worldwide. Considering this global issue, there is an urgent need to design and develop strategic measuring techniques with higher efficacy and precision to detect a broader spectrum of numerous contaminants. The development of precise instruments can further help in real-time and in-process monitoring of the generation and release of environmental pollutants from different industrial sectors. Moreover, real-time monitoring can also reduce the excessive consumption of several harsh chemicals and reagents with an added advantage of on-site determination of contaminant composition prior to discharge into the environment. With key scientific advances, electrochemical biosensors have gained considerable attention to solve this problem. Electrochemical biosensors can be an excellent fit as an analytical tool for monitoring programs to implement legislation. Herein, we reviewed the current trends in the use of electrochemical biosensors as novel tools to detect various contaminant types including toxic heavy elements. A particular emphasis was given to screen-printed electrodes, nanowire sensors, and paper-based biosensors and their role in the pollution detection processes. Towards the end, the work is wrapped up with concluding remarks and future perspectives. In summary, electrochemical biosensors and related areas such as bioelectronics, and (bio)-nanotechnology seem to be growing areas that will have a marked influence on the development of new bio-sensing strategies in future studies.

## 1. Introduction

Across the globe, the controlled or uncontrolled release of environmental contaminants, e.g., toxic heavy elements, antibiotics, and pesticides to the environment is a serious concern [1]. Therefore, there is an urgent need to design new prototypes to detect their presence in terrestrial and aquatic ecosystems. Low sample concentration and the lack of selectivity and sensitivity of traditional methods are among the significant bottlenecks of conventional methods. Moreover, conventional methods (e.g., chromatography) require long and specialized sample pre-treatment, which may potentially translate to time-consuming processes. In this context, electrochemical biosensors have proven to be useful tools to detect small sample volumes, low concentrations of biological components, and sometimes miniaturized analytical devices [2,3]. Recent advances in the fabrication and application of electrochemical biosensors for biomedical, agri-food, and environmental analyses have been reviewed [4]. Electrochemical-based techniques for sensing pollutants can be categorized as potentiometric, amperometric or coulometric, voltammetric (incorporating preconcentration and stripping steps), and conductometric. 

**Figure 1:**
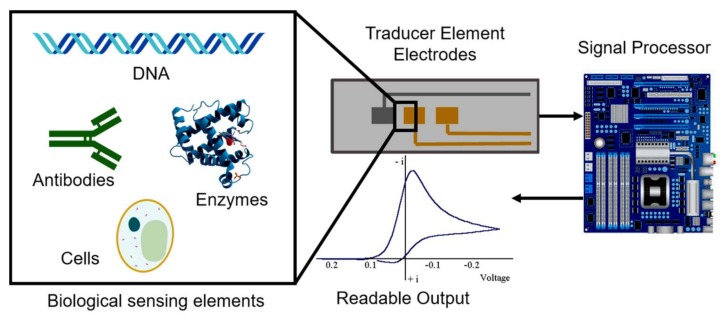
Scheme of an electrochemical biosensor. Biological sensing elements are coupled to electrodes. These traduce the signal to deliver a readable output.

**Figure 2:**
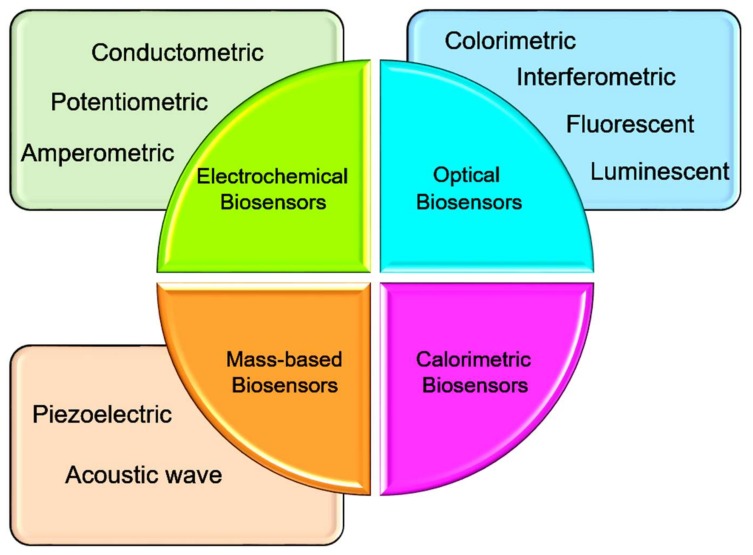
Four different classes and sub-classes of biosensors based on the type of transducer.

The ability to design highly specific recognition sites makes biosensors a suitable alternative to traditional chromatography-based methods [5]. Among existing biosensors, electrochemical biosensors have various advantages such as real-time monitoring, miniaturization, and the enhancement of selectivity and sensitivity. Also, electrochemical reactions deliver electronic signals, thus, it is not necessary to use complicated signaling elements. This facilitates the development of portable systems for clinical testing and on-site environmental monitoring [3]. Electrodes used in biosensors allow the conversion of biological signals into a readable output signal. The selectivity and sensitivity of these signals can be achieved via modification with specific biological elements such as DNA, enzymes, or cells (Figure 1). Figure 2 illustrates four different classes and sub-classes of biosensors based on the type of transducer. Based on the nature of the biological modification, electrochemical biosensors can be classified as either biocatalytic or affinity sensors. Electrochemical biocatalytic sensors are modified with biological elements able to recognize a target and induce a response of an electroactive molecule (e.g., enzymes). Meanwhile, electrochemical affinity sensors have a binding recognition element that releases a signal when it is coupled to the target (e.g., antibodies) [6]. 

There is much literature available on the utilization of biosensors for the detection of toxic heavy elements in the environment, including Pb, Cd, Hg, and Cu, and this has been reviewed elsewhere [7]. The current literature is lacking the implementation of electrochemical biosensors for the detection/monitoring of other environmental contaminants such as emerging contaminants (ECs). Due to the growing ECs concern, and the lack of selective and sensitive methods to detect them, different electrochemical biosensors have been developed in recent years. This review aimed to present the unique potential of electrochemical biosensors with particular reference to screen-printed electrodes and nanowire sensors and their role in the pollution detection processes. The first part of the review describes several ECs with reference to pharmaceutical and pesticide-based compounds, their occurrence and adverse outcomes. The second part majorly focuses on screen-printed electrodes and nanowire sensors. The development strategies and detection modes of electrochemical biosensors at large, and screen-printed electrodes, nanowire sensors, and paper-based biosensors, in particular, are discussed in the third section of the review. Towards the end, the work is wrapped up with concluding remarks and future perspectives.

## 2. Emerging Contaminants (ECs)

ECs are of growing environmental concern and comprise a variety of synthetic chemicals used in different industrial practices worldwide. ECs have been classified based on use, origin and/or effects. Potential sources include pesticides and herbicides, nanomaterials, phthalates, personal care products, additives to plastic, synthetic musk, brominated compounds, phytoestrogens, and pharmaceuticals (medication including hormones, pain relievers, antibiotics, etc.) [8,9]. Besides this wider spread, no or limited information exists about the regulation or precise analytical methodologies to determine the potential risk of ECs to ecosystems and public health [10]. Examples of EC categories derived from pharmaceutical compounds and pesticides as model sources are summarized in Table 1. 

The principal route of ECs is industrial wastewater which is known to have the highest concentration of pollutants [9,11]. Also, solids produced during the wastewater treatment have diverse contaminants and are used to fertilize the agricultural fields, urban parks, and residential yards [10]. Other compounds are discharged directly from industrial production processes into rivers or lakes and discharged chemicals move through the atmosphere and ocean currents. Ecotoxicological impacts have been reported for an array of synthetic chemicals of emerging concern. For example, endocrine disruption of fish reproduction, hormonal irregulation [12], renal failure in vultures by consumption of diclofenac [13], oxidative stress by engineered nanomaterials that damage the reproductive system of aquatic organisms [14], and inhibition of photosynthesis in algae caused by titanium dioxide nanoparticles [15]. All studies listed above were run in short-term exposure to the chemicals that might be considered as ECs. However, there is not enough evidence of long-term impact [16]. It is important to emphasize that ECs do not appear individually in the environment, which could lead to undesired synergistic effects [17].

ECs can also be divided according to their environmental impact, especially those have high solubility [8]. Two of the most important groups are pharmaceuticals (over-the-counter and prescription medication) and pesticides. When these compounds reach watersheds, their structures (isoforms) change. This represents a challenge for detection and quantification. Also, there is not enough evidence concerning the behavior of pharmaceutical products and toxicity to the environment [18,19]. The main detected groups of active pharmaceuticals ingredients in the world have been antibiotics, cardiovascular drugs, lipid regulators, antidepressants, and painkillers [18,20]. The lack of information about the persistence, bioaccumulation, and toxicity of most pharmaceutical substances on the market has raised the attention of researchers worldwide.

**Figure 3:**
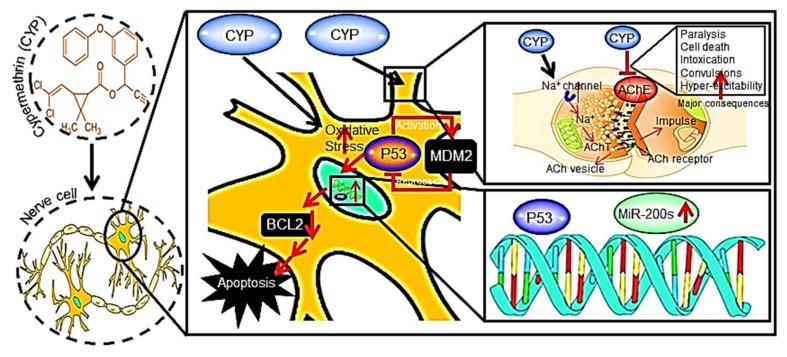
The neurotoxicity and other major consequences of CYP (Reproduced from Ref. [21] with permission from Elsevier).

Pesticides, e.g., cypermethrin (CYP), are being extensively employed in almost all sectors including agricultural, livestock, and households, etc. The toxicities induced by pesticides are assessed through different assays and models including in vitro, in vivo, or in situ strategies [21]. These have been detected in high concentrations in surface and groundwater [21]. The neurotoxicity and other significant consequences of CYP are presented in Figure 3. Pesticides are used worldwide to kill, incapacitate, or prevent pest damage to plants [16]. The concern for pesticides as ECs is due to their environmental persistence (half-lives) over time. Studies have proven that these can remain for a long time in solids and sediments. Also, these can be accumulated in non-human organisms with acute toxic effects, such as the mass killing of biota (e.g., bees, amphibians, and fish) [22,23]. Compounds, such as DDT, HCH, toxaphene, Aldrin, and dieldrin, are still present in soils and watersheds, even after being banned in 2002 at the Stockholm convention. There are not enough mechanisms to detect these chemical groups in water, and their potential for toxic effects on aquatic fauna remains unexplored [24,25,26].

## 3. Electrochemical Biosensors—Development Strategies 

Biosensor development depends on sensitivity, specificity, and parallelism. The approach to tackle this problem requires sensors to be produced in disposable materials of simple fabrication, have rapid responses, high accuracy, among others [30]. In order to develop biosensors with the mentioned requirements, a profound understanding of the detection mechanism is needed.

The most explored mechanisms currently developed involve Screen-Printed Electrodes sensors (SPEs) and Nanowire sensors. Other new paths include microfluidics and µTAS. However, this work focuses on SPEs and nanowire sensors since a high amount of work has been done with these strategies and a lot more can be done. The principles to design SPEs include; materials selection, screen selection, ink selection, substrate selection, and drying and curing stages. In the case of nanowires, the challenge is the right selection of an active material and its development into a nanostructure.

**Figure 4:**
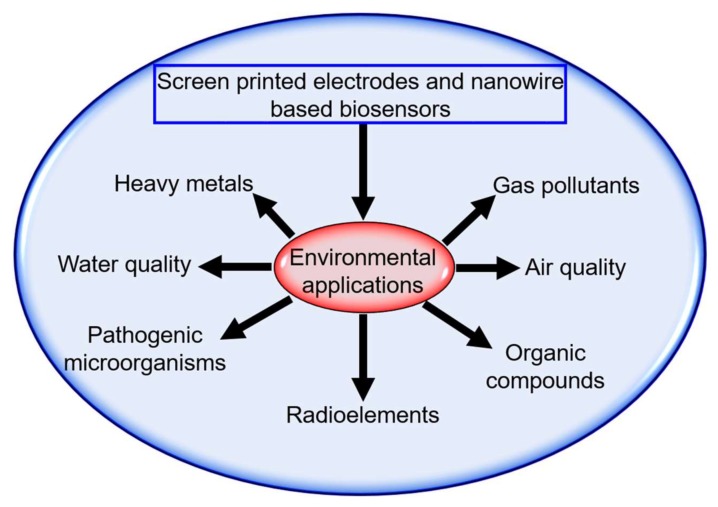
Environmental applications of SPEs and nanowire-based biosensors.

In both cases, the main idea is to have an electrochemical reaction from a detectable analyte and a sensor material that is transduced as voltage, current, and impedance which finally is processed and recorded by an electronic system [31]. The main topic of this section is about transducers as a detection mechanism for environmental application (Figure 4).

Transducers can be divided into pure electrical interfaces and electrical interfaces united with bio-receptors. The transduction mechanism, in general, is the electrochemical detection of current, charge accumulation, and conductive or impedance properties. This detection requires three electrodes i.e., (1) a reference, (2) a counter or auxiliary, and (3) a working electrode. The reference keeps a stable potential to compare with the working electrode and transduces the electrochemical reaction. The counter or auxiliary electrode establish a connection to the electrolytic solution and allow electron flow in the working electrode. Working and counting electrodes are conductive and chemically stable. 

The amperometric sensor measures the electron flow from the redox reaction at a constant potential or voltage. A basic amperometric sensor uses platinum for the working electrode and Ag/AgCl for the reference electrode. Besides, if a potential linear range is scanned, the peak value in current detected is proportional to the concentration of the analyte in the solution. Since not all analytes can be redox partners, mediators are used for this electrochemical reaction. Amperometric sensors have higher sensitivity than potentiometric sensors.

Potential sensors or potentiometric devices measure the voltage or charge potential at the working electrode compared to the reference one at the equilibrium. Potentiometric sensors establish the ion activity in the reaction creating a potential difference, which is described by the Nernst equation, also related to the electromotive force (Equation (1)).

(1) $$EMF\;or\;E_{cell}=E_{cell}^{0}-\frac{RT}{nF}\ln Q$$

where *E_cell_* represents the potential at equilibrium. *E*^0^*_cell_* is the constant potential of the cell, *R* is the universal gas constant, *T* is the absolute temperature in Kelvin, *n* is the number of the electron reaction, *F* is the Faraday constant, and *Q* is the ratio of ion concentration between anode and cathode [32]. The potentiometric devices offer stability since this mechanism does not chemically change the sample. However, the limit of detection for these types of sensors varies between 10^−8^ and 10^−11^ M [33].

Conductometric sensors measure the change in current flow provided by material in electrodes and media. Conversely, it is required to measure small changes in media conductivity to apply this method to low detection limits. Instead of doing direct measurements, the approach is set to immobilize complementary antibody-antigen pairs, enzymes, and DNA, among others to the electrode surfaces that lead to a real application of the conductive sensors [32].

Variations of the previously described mechanisms are cyclic voltammetry, chrono-amperometry and chrono-potentiometry, electrochemical impedance spectroscopy, and field effect transistor:

- Cyclic voltammetry is a periodic voltage variation measuring the change in current. The voltage variations can be performed in a wide range of patterns that lead to many forms of voltammetry. Some of the differences are polarography, linear sweep, differential staircase, normal pulse, reverse pulse, and differential pulse [6].
- Chrono-amperometry is a current measure of the steady state at a time when a square-wave voltage signal is applied to the working electrode. Besides, chrono-potentiometry is the voltage measured as a function of time while a constant or square-wave current is applied. In chrono-amperometry, the relation between current and analyte diffusion to the electrode is described by the Cottrell equation [31].
- Electrochemical impedance spectroscopy (EIS) measures the current response to an applied sinusoidal varying voltage. Exploring the frequency of the sinusoidal signal, it is possible to calculate the impedance as the real and imaginary components of the electrochemical system. EIS is a powerful tool since this technique evaluates the intrinsic material or system property of impedance, which is of high importance to biosensor development and applications [34].
- Field effect transistor (FET) is a configuration of a channel between two electrodes made of a semiconductor material and a transistor. The mechanism controls the electric field inside the channel along with its conductivity. A third electrode plays the role of the gate to drain the charge. This configuration allows the control channel to attract charge or repel it. In general, the array operates as a switch between conductive or non-conductive states when a drain-source voltage is higher than the gate-source and as an amplifier when it has constant current source given by the gate-source voltage. The FET technique is best used for applications with a weak signal and high impedance [35].

### 3.1. Detection Mechanism of Screen-Printed Electrodes (SPEs)

**Figure 5:**
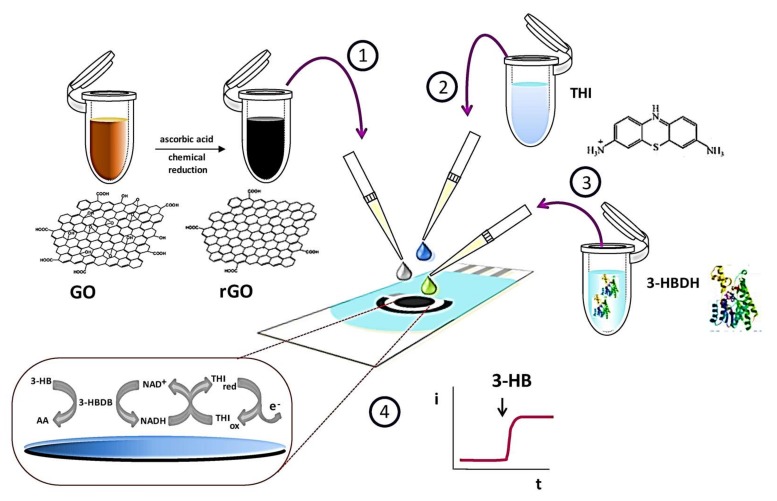
Scheme of the steps involved in the preparation and functioning of the 3-hydroxybutyrate dehydrogenase (3-HBDH)/thionine (THI)/reduced graphene oxide (rGO)/screen-printed carbon electrode (SPCE) biosensor. (Reproduced from Ref. [37], an open-access article distributed under the terms and conditions of the Creative Commons Attribution (CC BY) license (http://creativecommons.org/licenses/by/4.0/)).

SPEs are conductive ink printed circuits on a substrate. The technique uses a mold, stencil, or mesh to cast ink into a substrate by a physical barrier, and then the printed ink is cured and fixed with an insulator layer. The substrate materials include plastics, ceramics, paper, and most recently skin [36]. The conductive ink is desirable to be a good conductor but sometimes a material is required that interacts with the targeted analyte and previous works have used silver and carbon ink. Also, gold, platinum, noble metals, and other costly materials have been considered and tested. As said previously, SPE sensors relay on the redox reaction and its electrochemical consequences. The current properties to improve the sensing mechanism are based on; chemical modifications to increase catalysis, increase electrode surface by adding new morphologies, and stain electrodes with biomolecules to increase selectivity [30,36]. Martínez-García et al. [37] reported an electrochemical enzyme biosensor for 3-hydroxybutyrate detection using SPEs modified by reduced graphene oxide and thionine. Scheme of the steps involved in the preparation and functioning of the 3-hydroxybutyrate dehydrogenase (3-HBDH)/thionine (THI)/reduced graphene oxide (rGO)/screen-printed carbon electrode (SPCE) biosensor is illustrated in Figure 5. Amperometric responses were obtained according to the sequence of reactions shown in Figure 5 [37].

### 3.2. Detection Mechanism of Nanowire Sensors 

As said before, the challenge in nanowire sensors fabrication is the material selection. Then, the nanostructure fabrication technique, separated into two categories, “bottom-up” and “top-down”. In the first one, construction is performed by adding material sequentially to generate a chain of molecules in a controlled manner. In the second, a reduction is shown in the base material to create the structure [38]. The nanowires provide an excellent electron transport and optical excitation. The nanowires’ construction requires controlled dimensions, properties, and morphology. Therefore, the technology used is a solid-state crystallization with templates that include a nano-mold, anisotropic crystallographic structure, a liquid/solid interface to reduce symmetry during seed nucleation and tune growth by capping agents, more details can be found elsewhere [39]. The materials used to fabricate nanowires are carbon nanotubes, silicon, and conducting polymers.

## 4. Electrochemical Biosensors—A Solution to Pollution Detection

Researchers have dedicated many years of effort to the investigation and development of technologies towards the reduction/detection of the environmental impact of hazardous compounds. Several attempts have been made to explore electrochemical sensors potentialities to detect emerging contaminants including pesticides, antibiotics, heavy metals, perfluorinated compounds, etc. This further allows acquiring fundamental data on what type of contaminant is involved and under what capacity [40]. Electrochemical sensors and biosensors have been extensively proven to be helpful for the identification and analysis of specific compounds due to their simplicity, portability, and overall cost-effective manufacturing [41]. Table 2 summarizes optical biosensors and electrochemical biosensors used for rapid water contaminants e.g., toxic heavy elements detection. 

### 4.1. SPEs and Environmental Contaminants

A relatively significant issue for electrochemical sensors that utilize common, solid, and old-fashioned electrodes is the small surface area of the working electrode. They are usually designed to be miniaturized in order to provide the portability for in situ monitoring and compatibility with small amounts of sample. In other words, the size of the working surface area of an electrode in an electrochemical sensor is directly proportional to the detection efficiency of analytes [48]. A strategy for increasing electrode surface area of contact in electrochemical biosensors, independently of the application, is the use of nanostructured materials. Various materials at the nanoscale such as metallic nanoparticles, carbon-based nanomaterials (carbon nanotubes and graphene), carbon coatings, membranes, and even some conductive polymers have been used for increasing working electrode surfaces [70,71]. The screen-printing fabrication technique offers the potential to modify the morphology of the electrodes. For said purpose, a layer-by-layer deposition of ink on a solid substrate with a removable mesh or screen allows fabrication of novel designs with enhanced contact surface area. Consequentially, it leads to more efficient readings of analytes in samples. This technology also has advantages over conventional rod-shaped electrodes regarding flexibility, the possibility of process automation, reproducibility, the availability of many materials, and the cost efficiency of electrochemical substrates [30,37].

In recent years, graphene has been proven as an outstanding candidate for screen-printed electrodes fabrication due to its highly appealing mechanical, electrical, and chemical properties [72]. Low cost and innovative screen-printed graphene/carbon paste electrodes for electrochemical sensing of the most common electroactive analytes such as hydrogen peroxide (H_2_O_2_), nicotinamide adenine dinucleotide (NAD^+^), and ferric ferrocyanide (Fe(CN)_6_^3−/4−^) has been established. By using Ag/AgCl paste electrodes as a reference and PVC substrates, it was found that the addition of electrolytically exfoliated graphene to the carbon-based paste greatly enhanced the electrochemical responses of the screen-printed electrodes. Due to this addition of graphene, the optimum concentration of graphene in the carbon paste was determined to be 10%, at which the most efficient cyclic voltammetry (CV) was reproduced. It was also observed that the oxidation signals for each of the compounds were increased two-fold as compared with conventional screen-printed carbon electrodes [73]. 

SPEs have been extensively used for water quality tests (Table 3), and organic compound detection in environmental samples (Table 4). SPEs for the determination of toxic heavy metals including lead (Pb^2+^) and cadmium (Cd^2+^) has been reported and the collected data is summarized in Table 5. Researchers have used a mixture of graphene (G)/polyaniline (PANI)/polyestirene (PS) electrospun nanofibers. This screen-printed G/PANI/PS electrode was successfully proven to simultaneously detect Pb^2+^ and Cd^2+^ in real river water samples through anodic stripping voltammetry (ASV). It was observed that the increased surface area improved the electrochemical response of such electrodes due to the electrospinning technique for the acquirement of nanofibers, as well as the selection and mixture of materials [74]. The addition of graphene and other conductive elements into different materials in screen-printed electrodes has been shown to be very well applied to improvement in the detection of contaminants in the environment. It allows control monitoring of changes in the concentrations of such pollutants in various environments and therefore a more accurate treatment for such an issue [75].

### 4.2. Nanowire-Based Sensors and Environmental Contaminants

Another strategy used for increasing the surface-to-volume ratio, and thus augmenting the electrochemical sensitivity of sensors for the detection of analytes, is the implementation of nanowires for the assembly of various morphologies in electrodes. Such nanowires are often made of conductive or semiconductive materials such as gold (Au), silver (Ag), copper oxide (CuO), among others, although it depends on the analyte to be detected. Among the advantages that nanowires present over other materials are the simple preparation methods to obtain them, the surface-to-volume ratio, and high stability due to high crystallinity of such materials in their molecular structure, as well as high sensitivity and selectivity. The synthesis of nanowires can be classified into solution-phase growth and vapor-phase growth processes [119,120,121].

Copper oxide (CuO) nanowires were successfully attached to an electrochemical sensor, along with single-walled carbon nanotubes (SWCNTs) for the detection of organophosphorus pesticides used in the field of agriculture. Said electrochemical sensors composed of the aforementioned nanocomposite materials (CuO-SWCNTs), were proved to be highly stable and had good specificity towards malathion, as well as good selectivity against other pesticides, inorganic ions, and sugars. Since this work was proven in real liquid garlic samples, it demonstrates its applicability in selective detection of organophosphorus pesticides [122].

Nanostructured materials that include tin dioxide (SnO_2_) nanowires and zinc oxide (ZnO) nanorods have been designed for the sensing of gases such as ethanol, where the growth of such structures was controlled to make them perpendicular. The results revealed that the design of the hierarchical nanostructures enhanced their response to ethanol gas and the selectivity of the material for this gas, avoiding interference from gases in the environment such as ammonia (NH_3_), carbon monoxide (CO), hydrogen (H_2_), carbon dioxide (CO_2_), and liquefied petroleum gas (LPG). These enhancements are attributed to the improvement of homogenous and heterogeneous NW–NW contacts [123].

## 5. Paper-Based Electrochemical Biosensors

In the past few years, paper has become an increasingly common substrate for developing microfluidic devices and biosensors. Several paper-based materials hold several unique characteristics, i.e., porosity, liquid wicking rate, and fiber surface affinity to numerous analytes, etc. Moreover, paper-based models offer portable, on-site, and real-time monitoring, which are crucial for applications in different sectors including clinical, nutraceutical, and environmental [124]. Also, the broader spectrum of available supporting paper substrates, i.e., chromatography, filter, blotting, and office paper of different grades present their candidacy to engineer tunable devices and sensors and can be utilized to fulfill the requisite application [3]. Depending on the specific application needed, the fabrication strategy and analytical techniques of paper-based sensors can be tuned to fulfill the needs of the end-user [124]. Cinti et al. [125] combined the effectiveness of screen-printing with wax-printing to realize an all-in-one reagentless paper-based screen-printed electrochemical sensor to detect phosphate. They used a less-sophisticated filter paper to cover the laboratory bench and transferred the colorimetric reference method for the detection of phosphate in water directly into the paper support. A similar configuration was described by Medina-Sánchez et al. [126] in developing a lateral flow paper-based sensing device for lead and cadmium ions detection in environmental matrices. They screen-printed 500-µm wide electrodes, graphite ink formed the working and counter electrodes, while Ag/AgCl was printed as a reference electrode on a waxed chromatographic paper.

**Figure 6:**
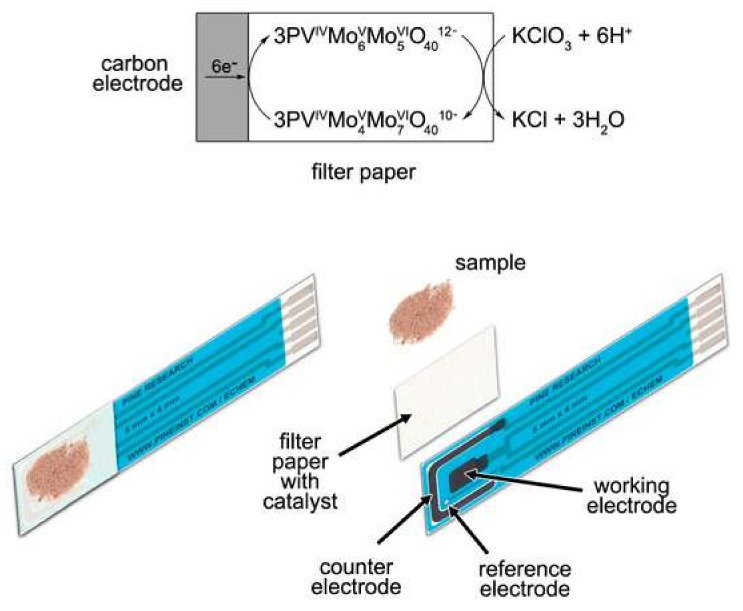
Paper-based electrochemical detection of chlorate. (Reproduced from Ref. [131], an open-access article distributed under the terms and conditions of the Creative Commons Attribution (CC BY) license (http://creativecommons.org/licenses/by/4.0/)).

Sample conditions and concentrations can fluctuate over the period of sampling and transportation back to the lab. Therefore, from the environmental application viewpoint, real-time and in-process monitoring of the generation and release of environmental contaminants such as heavy toxic elements and other hazardous pollutants is essential. Electrochemical biosensors are suitable options that allow on-site detection and accurate monitoring of environmental and separation conditions [124,127]. In this context, Nie et al. [128] developed paper-based electrochemical biosensor device for Pb(II) ion detection. Likewise, a simple, fast, and portable paper-based dual electrochemical/colorimetric system for simultaneous determination of gold and iron has been reported [129]. The challenge of whether to use a single drop of the solution or a stirred solution to accumulate the heavy metal was addressed by introducing a cellulose blotting pad as a sink for the outlet. This allowed continuous wicking of the solution across the electrodes which increased the efficiency and sensitivity of Pb(II) deposition during anodic stripping voltammetry [128]. Zhang et al. [130] prepared a three-dimensional microfluidic paper-based analytical device for the detection of toxic heavy metals, i.e., Pb^2+^ and Hg^2+^ in one paper working zone based on the potential-control technique. Moreover, the developed paper-based analytical tool was used for simultaneous detection of Pb^2+^ and Hg^2+^ in lake water and human serum samples, respectively. Very recently, Shriver-Lake [131] produced a paper-based probe by impregnating a vanadium-containing polyoxometalate anion, [PMo_11_VO_40_]^5−^ on carbon electrodes for the electrochemical determination of chlorate (Figure 6).

A multilayer paper-based device for colorimetric detection of Ni, Fe, Cu, and Cr and electrochemical detection of Pb and Cd is reported [132]. The colorimetric layer showed lowest detection limits for Cr, i.e., 0.12 μg, while electrochemical layer was highly sensitive with Cd and Pb detection limits as low as 0.25 ng. Subject to lowest possible detection and other critical parameters, new functionalities for paper-based sensors lead to lower limits of detection, simplified user operation, fluid flow control, signal amplification, component integration and new applications along with its low cost, portability, and simplicity features have been reviewed elsewhere [133]. Very recently, Cinti et al. [134] developed a fully integrated ready-to-use paper-based electrochemical biosensor for the assessment of nerve agent simulant (paraoxon) in real environmental sites. Paraoxon was linearly detected down to 3 µg/L. In this study, authors have used a carbon black/Prussian Blue nanocomposite as a working electrode modifier to improve the sensitivity of the paper-based screen-printed electrochemical biosensor device [134].

## 6. Concluding Remarks and Future Perspectives

In conclusion, it is clear from the data discussed above that the future of electrochemical biosensors will rely on the success of emerging sophisticated technologies, both at micro and nano level, along with in-depth contributions from electronics, materials science, biochemistry, and physics. The environmental pollution in different mediums is a serious health concern worldwide. Therefore, it is equally important to design and develop biosensor-based measuring techniques that can precisely detect various contaminants from a broader spectrum. However, biosensors for environmental analysis have several limitations that include (1) response time, (2) sensitivity, (3) selectivity, (4) compatibility, (5) affinity, (6) stability, and (7) lifetime, etc. These limitations should be eliminated for a successful on-site implementation as a competitive analytical tool. Besides the points mentioned above, it is also important to note whether the pollutant is gaseous (e.g., ozone, H_2_S, CO, O_2_, NOx, SOx) or whether it is confined to the solution phase. It is highly relevant to note that this type of technique in the design of such structures gives researchers the capability to experiment with the variations in the electrochemical responses of sensors with different structural arrays.

Owing to ever increasing public health concerns about the impact that environmental pollution may cause on the ecosystem, the demand for rapid detecting biosensor will increase in the near future. In spite of the past and current considerable research in electrochemical biosensor development, there is still a challenge to create improved and more reliable devices to avoid instrumental drift. In this context, in-depth study is needed to present the future trends in the biosensor field and other related areas such as bioelectronics, and bionanotechnology that will ultimately have a marked influence on the development of new bio-sensing strategies in the future. Nevertheless, the advent of “smart” and user-friendly electrochemical biosensors augers well for the future.

## Figures and Tables

**Table 1:** Examples of EC categories derived from pharmaceutical compounds and pesticides, detection techniques, and associated effects on human health and the environment.

Source	Examples of Main ECs	Distribution	Adverse Effects	Other detection Techniques	Reference
Pharmaceutical compounds	Fluoxetine (Prozac), Carbamazepine, Diphenhydramine Tetracycline, Erythromycin Sulfamethoxazole	Groundwater, surface water, wastewater treatment plant effluent, land applied biosolids, potable water, and recycled water.	Increased cancer rates, organ damage, Endocrine disruption, Antibiotic resistance in disease Environmental persistence Other Unknown health effects	Liquid chromatography coupled with mass spectrometry Gas chromatography	[8,16,27,28]
Pesticides	Organochlorine, Carbon-14 (^14^C)-labeled compounds, Organophosphorus, Pyrethroids, Carbamates, Triazines	Agricultural soil, groundwater, surface water, potable water, recycled water.	Damage to biodiversity and ecosystems health by the attack of non-target organisms, environmental persistence, pest resistance, Endocrine disruption	Liquid chromatography coupled to mass spectrometry	[16,25,28,29]

**Table 2:** Optical biosensors and electrochemical biosensors used for rapid water contaminants e.g., toxic heavy elements detection.

Sensing material	Contaminant	LOD	Working Range	Detection Time	Reference
Optical sensors					
Au NP	Pb^2+^	3 nM	3 nM to 1 μM	6 min	[42]
Au NP	Pb^2+^	100 nM	0.1–50 μM	25 min	[43]
GO QD	Pb^2+^	0.09 nM	0.1–1000 nM	20 min	[44]
Au NP	Hg^2+^	1 nM	1 nM to 1 mM	15 min	[45]
Au NP	Hg^2+^	9.9 nM	9.9–600 nM	10 min	[46]
Au NP	Hg^2+^	5 nM	50 nM to 10 μM	10 min	[47]
Au NP/RGO	Pb^2+^	10 nM	10 nM to 10 μM	few seconds	[48]
Au NP/RGO	Hg^2+^	25 nM	25 nM to 14.2 μM	few seconds	[49]
RGO	Hg^2+^	1 nM	1–28 nM	tens of seconds	[50]
SWCNT (no probe)	Hg^2+^	10 nM	10 nM to 1 mM	few seconds	[51]
CNT	Cd^2+^	88 nM	88 nM to 8.8 μM	30 min	[52]
SiNW	Pb^2+^	1 nM	1–104 nM	few seconds	[53]
SWCNT	*E. coli* DH5a	3 × 10^3^ CFU mL^−1^	3 × 10^3^–1 × 10^6^ CFU mL^−1^	20 min	[54]
Graphene	*E. coli* K12	10 CFU mL^−1^	10–10^5^ CFU mL^−1^	30 min	[55]
RGO	*E. coli* O157:H7	803 CFU mL^−1^	803–10^7^ CFU mL^−1^	25 min	[56]
Electrochemical biosensors					
Au	As^3+^ (1 M HCl)	0.26 nM	0.26–195 nM	100 s	[57]
Au–Pt NP	Hg^2+^ (1 M HCl)	0.04 nM	0.04–10 nM	100 s	[58]
Au NP/CNT	Hg^2+^ (0.1 M HClO_4_)	0.3 nM	0.5 nM to 1.25 μM	2 min	[59]
Carbon NP	Hg^2+^ (1 M HCl)	4.95 nM	4.95–49.5 nM	2 min	[60]
CNT	Pb^2+^ (1 M HCl)	0.96 nM	9.6–480 nM	180 s	[61]
Bi–CNT	Pb^2+^ (0.1 M acetate buffer)	6.24 nM	9.6–480 nM	300 s	[62]
MgSiO_3_	Pb^2+^ (0.1 M NaAc–HAc)	0.247 nM	0.1–1.0 μM	tenths of seconds	[63]
Graphene nanodots	Cu^2+^ (ammonium acetate solution)	9 nM	9 nM to 4 μM	15 min	[64]
MWCNT/GO	Pb^2+^ (0.1 M NaAc–HAc)	0.96 nM	0.96–144 nM	3 min	[65]
Graphene/nafion	Pb^2+^ (0.1 M acetate buffer)	0.096 nM	2.4–240 nM	300 s	[66]
Fe_3_O_4_/RTIL	As^3+^ (acetate buffer)	0.01 nM	13.3–133 nM	few min	[67]
Nanosized hydroxyapatite	Pb^2+^ (0.2 M HAc-NaAc)	1 nM	5.0 nM to 0.8 μM	10 min	[68]
Nanosized Co.	H_2_PO_4_^−^ (KH_2_PO_4_ solution)		10^−5^ to 10^−2^ M	1 min or less	[69]

**Table 3:** Some of the recently developed screen-printed sensors for water quality tests.

Analyte	Modifier	Detection Method	Reference
Liquids	Iridium and ruthenium oxide	pH sensor	[76]
Liquids	Phenanthraquinone moiety	pH sensor	[77]
Hydroxide ions	Nickel oxide bulk	pH sensor	[78]
Dissolved oxygen	CdS modified	Cathodic electrochemiluminescence	[79]
Nitrite	Poly(dimethylsiloxane)	Amperometric detection	[80]
Nitrite	Shallow recessed unmodified	Amperometric detection	[81]
Phosphate	Bisthiourea ionophores	Amperometric detection	[82]
Nitrite	Carbon Black	Multi-electrochemical methods	[79]
Phosphate	Electrocatalyst cobalt phthalocyanine	Amperometric	[83]
Phosphate	Cobalt phthalocyanine	Amperometric	[84]
Nitrate	Modified screen printed electrodes	Electrochemical detection	[85]
Nitrate	polymer (poly(vinyl alcohol)) modified	Amperometric	[86]
Nitrate	commercial screen-printed electrochemical cell	Amperometric	[87]

**Table 4:** Examples of the some of the recently developed screen-printed sensors for organic compounds detection in environmental samples.

Analyte	Modifier	Detection Method	Reference
Organophosphate	Poly(3,4-ethylenedioxythiophene) (PEDOT)	Amprometric	[88]
Organophosphate pesticides	Cobalt phthalocyanine	Chronoamperometry	[89]
Organophosphorus	Cysteamine self-assembled monolayer	Amperometric	[90]
Organophosphorus and Carbamate Pesticides	Unmodified	Amperometry, flow system	[91]
Aminophenol isomers	Untreated SPCE	Voltammetric	[92]
Organophosphorus Pesticide	Single-walled carbon nanotubes—Co. phthalocyanine	Amperometry	[93]
Organophosphorus Pesticide Dichlofenthion	Nanometer-Sized Titania	Photoelectrochemical	[94]
Herbicide isoproturon	Unmodified	Amperometric	[95]
Herbicide	Magnetic nanoparticles	Amperometric	[89]
Picric acid and atrazine	Self-assembled monolayer	Photo-electrochemical	[96]
Chlorsulfuron	Gold (Au) metal ions	Stripping voltammetry	[90]
Phenol and catechol	Bismuth nanoparticles	Amperometric measurements	[97]
Phenol and pesticide	Iridium oxide nanoparticles	Electrochemical measurement	[98]
Phenol	Carbon Black Paste	Amperometric	[99]
Phenolic compounds	Nano-HA-chitosan nanocomposite-modified gold electrode	Amperometric	[100]

**Table 5:** Selected and recently developed screen-printed sensors for heavy metal detections.

Analyte	Modifier	Detection Method	Reference
Pb^2+^ and Cd^2+^	screen-printed antimony and tin	anodic stripping detection	[101]
Cu^2+^	Macrocyclic Polyamine Modified Screen-Printed Electrodes	Square wave anodic stripping voltammetry	[102]
Cd^2+^, Cu^2+^	Diazonium modified electtrodes	Amperometric detection	[103]
Pb^2+^ and Cd^2+^	Bismuth-coated	Stripping voltammetry	[104]
Pb^2+^	Reduced graphene oxide	Square wave anodic stripping voltammetry	[105]
Zn^2+^, Cd^2+^ and Pb^2+^	Multiwalled carbon nanotubes	Differential pulse stripping voltammetry	[106]
Hg^2+^ and Pb^2+^	Polypyrrole/carbonaceous nanospheres	Square wave anodic stripping voltammetry	[107]
Pb^2+^ and Cd^2+^	Bismuth–carbon nanocomposites	Differential electrochemical methods	[108]
Pb^2+^	Bismuth-antimony film	Stripping voltammetric	[109]
Pb^2+^	4-carboxyphenyl-grafted	Anodic Square Wave Voltammetry	[110]
As(III)	Gold electrode	Sequential injection/anodic stripping voltammetry	[111]
As(III)	Nanoparticles	Linear sweep voltammetric	[112]
As(III)	Modified screen printed electrodes	Amperometric	[113]
Cd^2+^, Pb^2+^, Cu^2+^ and Hg^2+^ ions	Heated graphitenanoparticle	Electrochemical stripping	[114]
Hg^2+^	Gold nanoparticles-modified	Square wave anodic stripping voltammetry	[115]
Pb^2+^, Cu^2+^ and Cd^2+^	Mercury nano-droplets	Square wave anodic stripping voltammetry	[116]
Pb^2+^	Paper disk impregnated	One-step electrochemical detection	[117]
Cd^2+^	Nafion. Cd	Square Wave Anodic Stripping Voltammetry	[118]
